# Cognitive Control-Loop for Elastic Optical Networks with Space-Division Multiplexing

**DOI:** 10.3390/s21237821

**Published:** 2021-11-24

**Authors:** Silvana Trindade, Ricardo da S. Torres, Zuqing Zhu, Nelson L. S. da Fonseca

**Affiliations:** 1 Institute of Computing, State University of Campinas, Av. Albert Einstein 1251, Campinas 13083-852, Brazil; silvana@lrc.ic.unicamp.br; 2 Farm Technology Group and Wageningen Data Competence Center, Wageningen University and Research, 6700 AA Wageningen, The Netherlands; ricardo.torres@ntnu.no; 3 Department of ICT and Natural Sciences, Norwegian University of Science and Technology, 6009 Ålesund, Norway; 4 School of Information Science and Technology, University of Science and Technology of China, Hefei 230027, China; zqzhu@ieee.org

**Keywords:** virtual networks, space-division multiplexing, machine learning, control loop, cognitive networks

## Abstract

This paper introduces a new solution to improve network performance by decreasing spectrum fragmentation, crosstalk interference, blocking of virtual networks, cost, and link load imbalance. These problems degrade the performance of Elastic Optical Networks with Space-Division Multiplexing. The proposed solution, called Cognitive control loop (CO-OP), is capable of identifying a set of problems and creating plans to mitigate these problems. The CO-OP comprises four functions that employ learning algorithms to identify problems and plan a series of actions to reduce or eliminate them. The results show that the CO-OP can effectively decrease up to 30% the blocking of requests and up to 50% the crosstalk occurrence compared to existing algorithms.

## 1. Introduction

The increase in network traffic due to widespread bandwidth hunger applications has demanded network backbones with large capacity. The Wavelength-Division Multiplexing (WDM) technology, currently used in most optical backbones, allows bandwidth allocation only in a fixed amount (wavelength), which leads to inefficient use of the optical spectrum [1]. Moreover, these networks typically employ Single-Mode Fibers (SMF), whose capacity will be exhausted in the near future due to network traffic growth.

The Elastic Optical Networks (EONs) technology has been introduced a flexible bandwidth allocation scheme compared to that adopted in the WDM technology [1,2,3,4]. The EON allows the allocation of bandwidth at a fine granularity that matches the requirements of requests for lightpath establishment, avoiding bandwidth wastage.

Space-Division Multiplexing (SDM) has been employed in optical networks to overcome the looming fiber capacity crunch by enabling the use of space as an additional dimension [5,6]. Combined with the EON technology, SDM expands the spectrum band and maintains EON flexibility in bandwidth allocation.

Network virtualization allows the coexistence and isolation of Virtual Optical Networks over the same physical network. The Control Plane of Software-Defined Optical Networks (SDON) employs a centralized view of the network [7,8], which facilitates the optimization of resource utilization. The configuration of Virtual Optical Networks (VONs) initiates upon the arrival of a request, including the mapping of virtual nodes to physical nodes and virtual links to lightpaths [9,10,11,12]. Traffic patterns and resource availability determine the deployment of VONs [13,14], especially the route and spectrum allocated to lightpaths. Moreover, resource allocation needs to consider traffic fluctuation and impairment effect on transmitted signals [15].

The configuration of VONs is crucial for network operation and affects costs, blocking of requests, unbalanced link-load, bottleneck links, and impairing crosstalk. Few investigations [16,17,18] have addressed the deployment of VONs over EON-SDM but do not account for multiple factors [10,16,17,18,19], which may lead to network states far from optimal.

This paper proposes a framework, called Cognitive control loop (CO-OP), to optimize resource allocation for VONs over EON-SDM networks. The CO-OP considers a series of issues, such as spectrum fragmentation, crosstalk interference, blocking of requests, and link load imbalance. In CO-OP, the control plane is responsible for collecting information from the virtual and physical networks. The management plane is responsible for updating, processing, distributing, and storing information for the control loop function. The knowledge plane [20] comprises a control loop (monitor, analyze, plan, and execute functions) responsible for identifying and creating plans for the solutions to the identified problems.

In the control loop, the information is analyzed, and, if it identified a problem, a plan to address it is created and executed. We employ machine learning techniques to analyze and plan potential solutions. Using Reinforcement Learning (RL) to derive plans assures convergence to an optimized network state since RL adapts the plans, improving the solutions to the identified problems.

The results derived via simulation show that CO-OP can decrease blocking of VON requests up to 50% compared to that given by the employment of traditional methods. Moreover, it can decrease 30% the generated crosstalk, which impacts the blocking of requests.

The remainder of the paper is organized as follows. Section 2 describes related work. Section 3 describes the problem formulation of the virtual network configuration. Section 5 describes the CO-OP framework. Section 6 shows numerical results. Section 7 concludes the paper and describes future research directions.

## 2. Related Work

In VON configuration, each virtual node is mapped onto a physical node, and each virtual link onto a lightpath [10]. A VON can only be deployed if its resource requirement (i.e., transponders and frequency slots) can be allocated; otherwise, the request for a VON configuration is blocked (rejected).

The process of virtual link and node configuration reserves the requested amount of physical resources. If the physical network does not have enough resources to accommodate the VON, it is rejected.

VON configuration has been investigated to promote efficient support to Quality-of-Service. In Reference [10], a heuristic algorithm was introduced to configure VONs over SMF optical networks. The algorithm analyzes the centrality of the network topology for embedding a VONs on the physical network. Virtual nodes are mapped to physical nodes with the largest closeness value. The *K*-Shortest Paths (KSP) algorithm is employed to determine a path that has available resources to configure a virtual link between virtual nodes.

In Reference [21], the authors introduced an algorithm to enhance load balance among network links and reduce spectrum usage. Virtual nodes are sorted using their bandwidth and the computing resources availability, while physical nodes are sorted using the value of the Resource Contribution Degree (RCD) of the node, which impacts the allocation of resources of other nodes.

In References [16,17], a load-balancing algorithm was introduced for virtual network configuration on physical networks employing MCFs and Few-Mode Fibers (FMFs). In Reference [17], a constrained optimization model was introduced. It searches for the maximum index value among the unused slots across all links. A genetic algorithm was employed to solve the proposed formulation. In Reference [16], the authors introduced a procedure for the configuration VONs over SDM using FMFs. In the first step, the algorithm maps virtual nodes and links of an incoming VON request onto physical nodes and links; then, the algorithm verifies if the required QoS of all VONs is maintained in case o acceptance of the request. When evaluating a VON interference on the others, the Quality of Transmission (QoT) in the physical links plays a significant role.

An algorithm for virtual network topology adaptation using artificial neural networks was introduced in Reference [19]. Data is collected to allow the prediction of traffic variability so that VONs can be reconfigured onto another set of nodes and links to improve performance and resource utilization, which can lead to a reduction in the number of transponders needed.

In References [22,23], the authors proposed solutions for virtual network reconfiguration. In Reference [22], they used machine learning algorithms to predict traffic variability and the need for VON reconfiguration due to network congestion. However, their proposal does not account for networks employing SDM.

The authors in Reference [18] introduced two algorithms to cope with the spectrum fragmentation in lightpath establishment for VONs. The algorithms classify VON requests into immediate or advanced groups. To decrease the blocking of VONs, when slots are not available at the arrival time of a request, the establishment of a VON can be postponed to a future time, increasing the chances of accepting the request.

In References [24,25], the authors use machine learning to handle the spectrum fragmentation problem. In Reference [24], the authors employed a shortest path algorithm and reinforcement learning to cope with spectrum fragmentation and energy consumption in elastic optical networks based on single-core fiber [24]. They explored the container technology to increase the flexibility of virtual network mapping, as well as to provide fast deployment. The authors also proposed a new network architecture composed of a virtual network layer, network container layer, and physical network layer. Unsupervised learning algorithm is employed in Reference [25] to handle spectrum fragmentation in EON-SDM networks. When the fragmentation ratio reaches a limit, the spectrum is defragmented. The defragmentation algorithm employs k-Means algorithms to distribute the request on the spectrum to reduce fragmentation.

In Reference [26], the authors combined a distributed SDN control system with blockchain technology to improve the quality of services and resource utilization in elastic optical networks by reallocating the spectrum and reconfiguring VONs.

Most of previous investigations addressed networks based on single-core fiber [17,18,19,21,24,26,27] (Table 1). The space dimension in EON-SDM calls for more elaborate solutions, as well as open new opportunities to optimize resource usage. Moreover, existing solutions try to improve QoS by employing algorithms with high computational complexity.

Different from existing solutions that propose algorithms focusing on a single KPI (e.g., blocking of VON), we propose the CO-OP framework to support VON configuration taking into account several metrics, such as spectrum fragmentation, cost, link load balance, and crosstalk interference. Moreover, CO-OP creates plans to tackle multiple objectives rather than making decisions on a single metric. Furthermore, existing VON reconfiguration and spectrum reallocation solutions have not fully explored reinforcement learning as a solution to automate network management.

## 3. Statement of the Problem

This section describes the virtual and physical networks and the problem description [10] that CO-OP attempts to solve. The main objective of CO-OP is to increase the probability of virtual networks being accepted, as well as maximize the use of EON-SDM resources.

### 3.1. Physical Topology

The physical topology can be modeled as a graph G=(V,E), with *V* representing the physical nodes, and *E* representing the edges. A node v∈V has a limited number of transponders t(v) and CPU capacity c(v).

Physical links $e_{u,v}\in E$ connect a pair of nodes *u* and *v*, which have several frequency slots represented by the weight of the physical links. The *W* variable is the set of link weights, where $W=\{w\left(e_{u,v}\right)\;|\;e_{u,v}\in E\}$, and $w(e_{u,v})$ is the weight of the link $e_{u,v}$. The weight of a link $w(e_{u,v})$ is the sum of available slots between nodes *u* and *v*.

The available capacity of a physical node $R_{V}\left(v\right),v\in V$, is defined by $R_{V}\left(v\right)=c\left(v\right)-\sum_{c}\left(v\right)$, in which x↑y means that the virtual node *x* is mapped onto physical link *y*. The available bandwidth in a physical link $R_{E}\left(e_{u,v}\right)$ is defined by the total bandwidth available in the physical link $e_{u,v}\in E$, $R_{E}\left(e_{u,v}\right)=b\left(e_{u,v}\right)-$. The available bandwidth of a path p∈P is then given by $R\left(p\right)=min_{e_{u,v}\in p}R_{E}\left(e_{u,v}\right)$.

Table 2 shows the notation used in the paper.

### 3.2. Virtual Topology

The ith request for the establishment of a VON with lifetime $t(G_{v}^{i})$ is modeled by a graph $G_{v}^{i}=\left(V_{v}^{i},E_{v}^{i}\right)$, where $V_{v}^{i}$ and $E_{v}^{i}$ represent the set of virtual nodes and virtual links of the ith request, respectively. Each virtual node $v_{v}^{i}\in V_{v}^{i}$ is associated with a computing resource requirement *c*, while each virtual edge $e_{v}^{i}\in E_{v}^{i}$ is associated with a bandwidth requirement $bw(e_{v}^{i})$.

A virtual network can be accepted only after its nodes and links have been mapped to the physical network G=(V,E). Resources are reserved for the entire lifetime of the VON, $t(G_{v}^{i})$. Physical resources are shared among VONs. When the lifetime of a virtual network comes to an end, the allocated physical resources are released and can be used by other VONs.

In the traditional VON allocation problem, the solution is mapping virtual nodes to physical nodes and virtual links to physical paths [28,29]. In the specific case of the configuration of VONs over EON-SDM, we need to consider EON-SDM the issues that might degrade performance, such as spectrum fragmentation.

The unique aspects of CO-OP are the combination of information about the states of the physical and virtual networks, and the consideration of past decisions in future ones. Moreover, CO-OP is capable of identifying multiple problems in the network that can cause performance degradation and creating a plan to handle them.

## 4. Metrics Used

This section shows the metrics used as features in the algorithms employed in the CO-OP framework.

To measure the dispersion of the network load, we use the standard deviation $S_{D}$ of the loads on network links [21]:

(1) $$S_{D}=\sqrt{1/\left(N_{l}-1\right)\sum_{l_{i,j}\in E}{\left(\max\{|F_{l_{i,j}}\left|\right\}-\mu_{s}\right)}^{2}},$$

(2) $$\mu_{s}=\sum_{l_{i,j}\in E}\max\{|F_{l_{i,j}}\left|\right\}/N_{l},$$

where $\mu_{s}$ represents the maximum index of a frequency slot used in links, and $N_{l}$ represents the number of physical links. A network with a smaller $S_{D}$ value indicates a satisfactory load balancing performance.

We use the *long-term revenue to cost ratio* to evaluate the efficacy of resource utilization [10]. The revenue denotes the economic benefits of accepting a virtual network request and the cost denotes the physical resources allocated to virtual networks. The revenue can be calculated by summing all virtual resources accepted over time, as follows:

(3) $$R\left(G_{v},t\right)=\sum_{e\in E_{v}}bw\left(e^{v}\right)+\sum_{n\in N_{v}}c\left(n^{v}\right),$$

with $G_{v}$ being the virtual network, *t* the time, $bw(e^{v})$ and $c(n^{v})$ the bandwidth required by a virtual link *e*, and *n* the computing resource of a virtual node.

The *long-term average revenue* is defined as [10]:

(4) $$\lim_{T\to \inf}\frac{\sum_{t=0}^{T}R\left(G_{v},t\right)}{T}.$$

The *cost of the request* $G_{v}$ at time *t* is defined as [10]:

(5) $$C\left(G_{v},t\right)=\sum_{p\in P(G_{v})}h\left(p\right)\times bw^{s}\left(p,G_{v}\right)+\sum_{n\in N_{v}}c\left(n^{v}\right),$$

where $P(G_{v})$ is the entire set of physical paths allocated to the virtual links in $G_{v}$, h(p) is the number of hops in a path *p*, and $bw^{s}\left(p,G_{v}\right)$ is the reserved bandwidth over a path. The long-term average cost is defined as [10]:

(6) $$\lim_{T\to \inf}\frac{\sum_{t=0}^{T}C\left(G_{v},t\right)}{T}.$$

The long-term revenue to cost ratio is given by [10]:

(7) $$\lim_{T\to \inf}\frac{\sum_{t=0}^{T}R\left(G_{v},t\right)}{\sum_{t=0}^{T}C\left(G_{v},t\right)}.$$

The *fragmentation ratio* is defined as the mean ratio between the isolated and small-sized blocks of slots and the total number of slots in the spectrum [4,30]. These isolated blocks of slots are neither contiguous in the spectral domain nor aligned along the fiber links, making it difficult to allocate them for a lightpath.

For the *network fragmentation ratio*, the routes of source-destination pairs represent the contiguous aligned available slot ratio in the network. The contiguous and aligned available slot ratio is defined by $\phi =\sum_{d\in D}\sum_{k\in K_{d}}w_{dk}\cdot \psi_{dk}$, where $\psi_{dk}=\gamma_{dk}/Z$ is a weight proportional to the traffic load of route $k\in K_{d}$ of source-destination pair d∈D, where $\sum_{d\in D}\sum_{k\in K_{d}}w_{dk}=1$. *Z* is the number of spectrum slots in each link, assuming that all links have the same number of slots. *D* and $K_{d}$ are the set of all the source-destination pairs, and the set of routes of source-destination d∈D. $\gamma_{dk}$ is the maximum number of contiguous aligned available slots for route $k\in K_{d}$ of source-destination pair d∈D. The fragmentation is then defined as χ=1−ϕ [30].

The *closeness centrality* [31] expresses how close a node is to all other nodes in a network. It can be calculated as follows:

(8) $$C_{C}\left(\upsilon \right)=\frac{n-1}{\sum_{\upsilon \neq \nu }d\left(\upsilon ,\nu \right)},$$

where $C_{C}\left(\upsilon \right)$ is the closeness centrality of the node υ, and d(υ,ν) the shortest distance between nodes υ and ν.

In EON-SDM networks [32] with multiple core fibers with cores arranged in a hexagonal array, the following equation gives *mean crosstalk* (XT) interference:

(9) $$XT=\frac{c-c\times \exp(-(c+1)\times 2\times h\times L)}{1+c\times \exp(-(c+1)\times 2\times h\times L)},$$

“where *c* is the number of adjacent cores, e.g., for seven cores, *c* is equal to six for the center core, while it is equal to three for all the other cores; *L* the length of the fiber, and *h* the mean increase in crosstalk per length unit calculated by” [32]:

(10) $$h=k^{2}\times R/\left(\beta \times \Lambda \right),$$

“where *k* is the coupling coefficient, *R* the bending radius, Λ the core pitch of the lattice, and β a propagation constant” [32]. The crosstalk interference is proportionally affected by the number of adjacent cores and the length of the fiber.

## 5. CO-OP Framework

This section introduces the Cognitive control loop (CO-OP) framework based on cognitive networks [33,34,35,36,37]. This framework is designed to tackle problems that occur in the operation of EON-SDM networks, such as spectrum fragmentation, crosstalk interference, and link load imbalance. The CO-OP framework is a control loop that uses functions to analyze and create plans comprising actions to handle impairments in offering the required QoS and QoT.

Using a cognitive control loop concept based on monitor, analyze, plan, and execute functions, we can separate the process of finding a problem and creating a solution to it. Moreover, it allows us to automate the (re)configuration process, ensuring the target metric values are achieved.

The CO-OP framework uses supervised and reinforcement learning algorithms. The monitor function collects a set of metrics from the physical network and virtual topologies to identify the occurrence of potential problems. The problem identified is notified to the analyze function to determine its criticality by using a supervised learning algorithm. Such classification is then used by the plan function, which uses reinforcement learning to generate an optimal plan to mitigate the problem. The execute function coordinates the execution of the plan on the control plane and then verifies if the proposed changes were sufficient to solve the identified problem.

**Figure 1:**
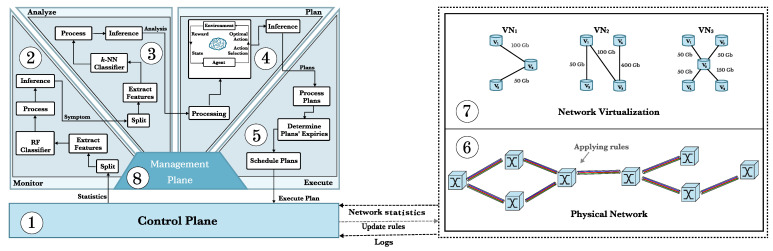
The structure of CO-OP framework in EON-SDM networks.

Figure 1 illustrates how the CO-OP framework is used to improve the configuration of VONs. The CO-OP continuously monitors the physical and virtual networks to capture data to help identify existing problems. The centralized control plane provides statistics on specific metrics. The collected data is stored in the management plane and used by the MAPE functions and the control plane. The objective of the CO-OP framework is to improve the configuration of the network resources.

In our framework (Figure 1), the monitor (2) uses a control plane (1) to obtain statistics from the physical network (6) and the virtual networks (7). We collect the fragmentation ratio, available transponders ratio, link load, available spectrum resources, blocking ratio, resource usage and mean crosstalk from the physical network. Moreover, we collect the mean bandwidth, blocking ratio, and the mean resource demand from the virtual networks. The monitor is able to detect whether the current network state indicates the existence of any operational problem. If a problem is detected, CO-OP tries to identify the problem in the next function (3). The monitor function is able to detect whether the current network state indicates the existence of an operational problem. If a problem is detected, CO-OP tries to identify the problem in the next function (3).

The problem is then handed over to the analyze function (3), that tries to identify the source of the problem. For that, the analyze function uses a supervised machine learning algorithm. The result of the analysis is passed to the plan function (4). The plan function also employs a machine learning algorithm to propose an optimal plan of changes based on the criticality of the identified problem. The plan is handed over to the execute function (5), that will coordinate the execution of the plan on the control plane (1). Lastly, the management plane (8) is updated with information derived in each step and information on the success of the execution of the plan.

### 5.1. Monitor Function

The monitor function periodically collects statistics from a centralized control plane and tries to associate these statistics to symptoms (problems) that are later analyzed. The monitor plane collects the following set of statistics received from the control plane:

(i) link-load distribution,
(ii) available slot ratio,
(iii) long-term revenue to cost ratio,
(iv) network fragmentation ratio,
(v) mean transponder utilization,
(vi) mean crosstalk interference,
(vii) mean computing resource utilization,
(viii) spectrum fragmentation degree (number of links that have high fragmentation, and
(ix) mean number of lightpaths per link.

The CO-OP framework determines the occurrence of a problem in the network operation based on the statistics collected by the control plane.

The CO-OP framework employs learning techniques o identify the existence of problems. Using supervised learning techniques, it is possible to identify problems and comprise solutions that can immediately mitigate the problem. In the monitor function, the input is a set of statistics, and the output generated by the algorithm is a potential problem limiting the achievement of target network efficiency.

In the CO-OP framework, the monitor function can identify the following problems: high cost, spectrum inefficiency, traffic load unbalance, and link overload. If a problem is identified, it is passed to the following function. If there is no problem, the algorithm stores the generated information for the support of future decisions.

### 5.2. Analyze Function

The objective of the analyze function is to characterize the problem, generating enough information to create a plan.

The analyze function is used to determine the criticality of the problem detected by the monitor function. The criticality gives the extent to which a problem interferes with the resource allocation to other VONs.

The criticality of a problem can be classified as low, medium, or high, depending on the risk of compromising the network performance.

A low classification means that the problem is not interfering with the network performance at that moment, which could also indicate that the problem has recently begun. A medium classification means that the problem is impacting the network performance, but not to a significant extend. A high classification means that the identified problem is causing significant degradation of network performance. CO-OP employs a supervised learning algorithm to classify identified problems, chosen among those in Table 3.

After classifying a problem, the analyze function provides the information on the locality of the problem, if it is a local or global problem. A local issue means that few links or nodes are suffering from the identified problem. On the other hand, a global issue means that most links or nodes suffer from the identified problem.

The analyze function uses a supervised learning algorithm to classify each node or link. The statistics used by this function include link fragmentation ratio, number of transponders used in each link, bandwidth utilization for each virtual link, number of slots used, crosstalk, number of virtual links configured in each physical link, blocking of lightpath establishment in each physical link, and centrality measures (e.g., closeness centrality). All measures must be correlated in order to mitigate the problem.

For local problems, the analyze function investigates which elements (nodes or links) are causing a problem. A collection of nodes may cause a problem that propagates globally. It is also possible that a single node or link is so severely affected that it alone causes changes in the global network operation.

If the resources used are low, and the associated cost is high, the cost yielded from the acceptance of a VON request is non-justifiable. The cost associated with a node is directly proportional to its links utilization. To evaluate the criticality of the cost problem, the analyze function of CO-OP requires information on the used number of transponders in nodes, bandwidth usage, the blocked number of requests due to limitations in physical nodes, and the closeness centrality of physical nodes.

In order to evaluate spectrum usage in each link, the analyze function needs to know the crosstalk generated, the number of virtual links that are using the physical link, and the total number of slots required by each virtual link. This metric indicates whether high crosstalk is either the underlying cause of a problem or the link under a heavy load without causing any adverse effect. For example, when a link has high crosstalk, and the number of virtual links using the physical link is small, the allocation algorithm could fail, so the classification is high. Notice that sometimes only two metrics are necessary to classify a problem, but, for complex problems, multiple metrics are necessary.

In order to evaluate the relevance of some links to the network operation, the analyze function needs to know the loads of the links, the number of virtual links that are using this physical link, and the sum of the closeness centrality values from the source and destination nodes associated with this link. Nodes with high closeness centrality values impact the disjointedness of paths and consequently favor the formation of bottleneck link, as well as the blocking of requests.

In order to evaluate the relevance of overload links, the analyze function needs information on the loads of the links, the number of virtual links that are using each physical links, the crosstalk generated in the available slot ratio per link.

If most of the links/nodes were categorized as high, then the problem is considered global. After this process, it informs the analysis to the following function (plan), which will create the new rules to mitigate the existing problem.

### 5.3. Plan Function

The plan function will comprise plans for the adjustments necessary to enhance the efficiency of EON-SDM networks based on the results provided by the analyze function. A plan is defined as a collection of procedures that a centralized control plane will execute to ameliorate the identified problem. These procedures can limit the resource usage of the network. The plan function selects procedures to enact the desired alterations done by the control plane. The aim is to gradually improve the policy used until an optimal, or near-optimal policy is reached.

**Figure 2:**
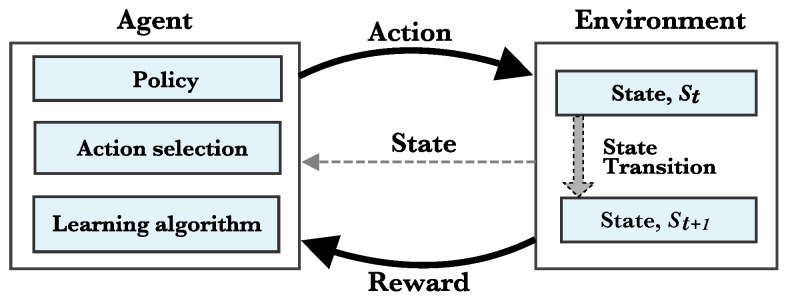
Agent-Environment interactions in the RL.

In order to create a plan, the CO-OP framework employs the Reinforcement Learning (RL) algorithm (Figure 2) *Q*-Learning [38] in the plan function since it does not require a model of the environment. A plan is a set of changes to be employed in the network. The basic idea of RL is that an agent can learn from a series of successful and failed actions based on the received reward for the actions made.

The main advantage of using *Q*-Learning is the possibility of dynamic composition of plans that are possible by rewarding the last plan and identifying the effect produced. The goal of using *Q*-Learning is to compose a plan with actions that can mitigate the problem of the network based on exploration and feedback from past experiences. Without the use of RL algorithms, a static plan could increase the problem, potentially generating additional problems or simply making useless changes.

The RL algorithm identifies the optimal plans considering the past decisions, and the rewards received. By employing RL in the CO-OP framework, we are able to decrease the occurrence of most of the issues described in Section 5.1. If a plan turns out to be not much effective, CO-OP will learn and adapt.

*Q*-Learning algorithm is one of the most popular algorithms to determine *Q*-value functions in model-free approaches [39,40]. “*Q*-Learning is exploration-insensitive, meaning that it will converge to the optimal policy regardless of the exploration policy followed, under the assumption that each state-action pair is visited an infinite number of times, and the learning parameter α decreases” [38,39].

In *Q*-Learning [40,41], the policy is composed of a *Q*-value, Q(s,a) for each of the possible state-action combination, where Q(s,a) denotes the value of doing action *a* in state *s*. After each learning episode, the *Q*-values are updated as follows:

(11) $$Q_{k+1}\left(s_{t},a_{t}\right)=Q_{k}\left(s_{t},a_{t}\right)+\alpha \left(r_{t}+\gamma \max_{a}Q_{k}\left(s_{t+1},a\right)-Q_{k}\left(s_{t},a_{t}\right)\right).$$

“The agent makes a step in the environment from state $s_{t}$ to $s_{t+1}$ using action $a_{t}$ while receiving reward $r_{t}$. The parameters 0⩽α⩽1 and 0⩽γ⩽1 are referred to as learning rate and discount factor, respectively” [40,41].

The reinforcement learning agent usually has two possible strategies for how actions are selected:

(i) exploit the knowledge found for the current state s∈S by taking action a∈A that maximizes Q(s,a), or
(ii) explore by selecting a different action from the one that is currently considered as the best one, with the objective of trying to learn if there is a better action than the chosen one.

In order to learn, it has to explore but to perform well, it should exploit what it already knows.

“The ϵ-greedy action selection is used, ϵ gives the probability for a random action to be chosen. The actions are selected by their corresponding *Q*-values” [41].

Every identified problem can have a possible action to be executed in the network, where the network is the environment. The agent navigates to create an action. The resulting plans can comprise multiple changes depending on the *Q*-Learning algorithm.

The state-space, action space, and reward function are designed as follows. The state-space *S* is represented by the problem evaluation given by the monitor and analyze functions, i.e., the problem which the network faces.

The action space is represented by the possible changes that can be added to a plan to mitigate the identified problem. We define the action space as A={0,1}, where 1 represents that a change will be added in the plan, and 0 otherwise.

The reward function evaluates if the objective of each action was achieved. The reward function is calculated based on the action; each plan has its own reward function. Result values vary between −1 and +1. To achieve the objective associated with each problem, we developed mechanisms to evaluate the plan. We designed the reward function as the estimator of a plan to mitigate a problem. The control plane is responsible for collecting the reward results and informing them to the management plane. The management plane communicates with the plan function to improve the learning algorithm for future plans.

The agent may decide to do nothing or even decide to promote global changes, such as defragmentation.

The complexity of the *Q*-Learning algorithm is O(ln|A|), where *A* is the set of actions that could be chosen for handling an existing problem.

### 5.4. Execute Function

After a plan is generated, a series of rules need to be established in order to reward the plan. In Table 4, the first column represents the procedures that the control plane will execute (plan function), the second column gives where this procedure will be executed, the third column the associated problem (monitor function), and the last column the criticality of the problem (analyze function).

The plan generated is sent to the execute function which will carry it out. Each action in a plan has a specific order to be executed in such a way that the result of one action does not impact changes of another action.

One of the possible actions is a redirection that changes the mapping of a virtual link to physical links on a route different than the used one. The redirection plan aims at reducing the load on the links to a specific value. The plan indicated the remapping of all virtual links mapped onto specific physical links. If the target load cannot be reached, the information is passed to the management plane so that the reward value can be updated accordingly.

The blocking action temporarily forbids a physical node or link from being allocated to a new VON request. The control plane continuously verifies if the condition to unblock physical nodes and links is satisfied. To unblock a physical link, it must have a number of available slots equal or greater than the mean available slots, and the link load must be lower than the mean link load. The used computing resource must be equal or lower than the mean computing resource utilization to unblock a node. In addition, the number of transponders used must be lower than the mean number of transponder used.

The allocation limiting action temporarily limits the resources of a physical node (transponders) or physical link (spectrum). By doing this, it will force other links and nodes to be used for configuration, thus balancing the load on the network links. The control plane removes the limit of a physical link usage when the lifetime of one or more VON requests expires and the resources are released. It verifies if the link load and available slot ratio decreased after the plan was executed. The mean crosstalk value must be lower than the mean crosstalk on the network links.

The reward for limiting actions is only informed when the limitation is removed, at which point it is possible to see if the action was successful or not. As VONs expire and resources are released, we evaluate if the goals for the limitation are reached, and, if not, the reward is decreased. The reward starts positive, but as more VONs expire and the goals are not reached, the reward will accumulate negatively. When the limitation goals are reached, the limitation is finally removed, and the accumulated reward is informed to the management plane so the learning algorithm can learn from this feedback.

In order to execute the actions of a plan, a priority ordering for execution needs to be defined. The redirection plan is only executed after blocking and limiting actions if they are present on the same plan, in order to guarantee that plan does not change something that impacts another.

After an ordering is established, the execute function is responsible for informing the changes to the control plane. The execute function also informs the control plane about the verification it needs to periodically do in order to determine whether restrictions can be removed or not.

### 5.5. Management Plane

The management plane comprises a database, where all relevant data related to CO-OP is stored, including analysis, policies, plans, and statistics. This plane has information on the state of virtual and physical network topologies. The management plane share data with the MAPE (monitor, analyze, plane, execute) functions.

The MAPE functions generate the data stored and the information sent periodically by the control plane. The plans are executed by the control plane, and the information collected during the execution is informed to the knowledge plane. Some actions, such as the reconfiguration of lightpaths, do not need to be verified periodically. The node blocking rule is verified whenever the lifetime of a VON using that node expires and the resources are released.

**Figure 3:**
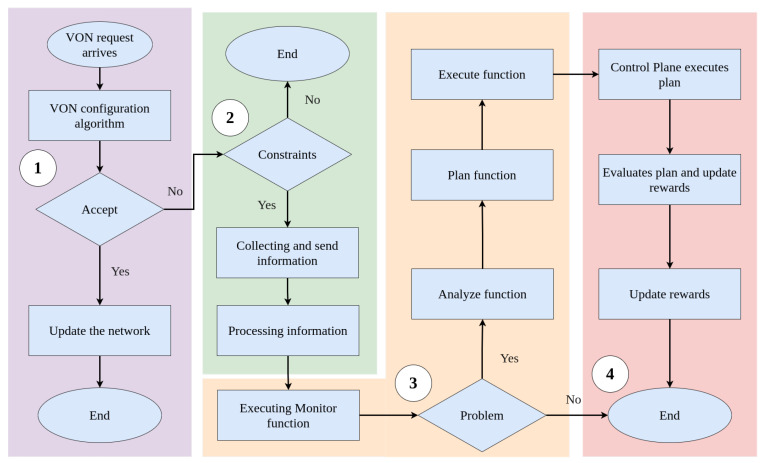
Illustration of how the CO-OP, control plane, and VON configuration algorithm are integrated.

Figure 3 shows the flowchart of the proposed solution, including the integration of CO-OP, the control plane, and the VON configuration algorithm (Step 1). In this figure, whenever a VON request is rejected due to resource limitations (Step 2), then the CO-OP framework starts to work (Step 3). All steps of CO-OP are executed when the monitor function identifies a problem (Steps 3 and 4).

## 6. Performance Evaluation

CO-OP employs supervised learning techniques to learn about potential problems in the operation of EON-SDM networks. Table 3 shows a comparison between different supervised algorithms for the monitor and analyze functions. The assessments of the accuracy of these algorithms were conducted independently. To estimate the accuracy of the analyze and monitor functions, we employed a synthetic dataset produced from the simulation of networks with different topologies (available at CO-OP framework repository, accessed on 2 November 2021). Before the network simulation, we train each model with the same set of features provided by the synthetic dataset for the monitor and analyze functions, as well the learning algorithms, and then we calculate the accuracy measure based on the training results (Table 3). For example, considering the monitor function, during the network simulation, a set of data (Section 4) are collected and normalized; after that, a sub-set of features provided by the dataset is used as the input for learning algorithms (Table 3). To select the features, a feature selection approach is employed, and then these selected features are used to train the data for all different models.

The codes of the algorithms in Table 3 are available in the Scikit-learn library [42] and the code for the *Q*-Learning algorithm in the Reinforcement Learning and Planning (BURLAP) library [43]. The classification in the monitor function classifies the current state of the network as either problematic or normal. The problems identified by the monitor function can be spectrum inefficiency, high cost, and unbalanced load. The analyze function tries to understand the criticality (low, medium, and high) of the identified problem. The supervised algorithms must be trained before being employed to detect problems. The training process aims at reaching accurate classifications.

For the training process of the monitor and analyze functions, we used two synthetic datasets, each having $10^{3}$ samples. The samples were collected from simulations using a greedy VON configuration algorithm and the NSFNET and USA topologies [44]. The greedy VON algorithm does not use any optimization approach to allocate VONs. When a VON request arrives, the algorithm allocates the requests in the first available node/link with sufficient resources. Samples comprise several characteristics (features) that define the network state in a period, including the statistics and raw data (described in Section 5.1 and Section 5.2), from virtual and physical networks.

After the dataset was built and the values normalized, outliers were removed and features selected. Considering supervised algorithms, a feature selection algorithm was employed to reduce the number of features, which is one of the most critical pars of learning processes since the number and subsets of features have substantial impact on the learning process. These features need to represent the selected problems, and they directly impact the model accuracy. For the data collection, we employed feature selection to define a group of features that optimally represent the patterns of each problem. We employed a correlation approach to detect relevant features in order to comprise the dataset for training. We used https://pandas.pydata.org/ (accessed on 2 November 2021) the Pandas libraryto derive correlation matrices and heatmaps to the selected features. For the analyze function, the selected features were acceptance ratio, blocking ratio, mean bandwidth required for each VON, and mean resource usage per VON, blocking ratio, link load, available spectrum resources, available transponders ratio, mean crosstalk, compute resource usage, and fragmentation ratio.

After performing feature selection, the final datasets are used to train the *k*-Nearest Neighbor (KNN), Support Vector Machine (SVM), Neural Network (NN), Naive Bayes, Random Forest, and Logistic Regression algorithms. The number of correct classifications, the accuracy, and the errors in numeric predictions were used to evaluate the performance of these algorithms. The values of accuracy obtained in the evaluation are shown in Table 3.

The KNN algorithm employed in the analyze function was configured with k=3, where *k* is the number of possible criticality classifications considered in this paper. The Neural Network obtained the best accuracy with five layers, considering at most 6 layers. Cross-validation adapting for different portions of the dataset was used to increase accuracy, and 10% to 50% of the dataset was used for this purpose.

For the monitor function, the learning algorithm returns an existing problem in case it is detected. We performed tests with all the learning models since, in the CO-OP framework, the result given by one algorithm may impact the result given by another. Evaluating their interaction with reinforcement learning is crucial for the choice of the best combination of algorithms to be used.

For comparison purposes, we employed the Key-Link and Resources Contribution Degree for VONs Mappings (KLRVM) algorithm [21], both with and without the CO-OP framework. For lightpath selection in KLRVM, we used the *K* Shortest Path Algorithm (KSP) (with k=3). For the spectrum selection, we used different modulation formats. The First-Core First-Fit (FCFF) algorithm was adopted since it is one of the most popular algorithms in the literature.

As previously mentioned, the CO-OP framework monitors the network to detect problems and create plans that can ameliorate these problems, but it does not configure VON requests. Although the KLRVM algorithm was employed for this purpose, the CO-OP can work with any VON configuration algorithm.

**Figure 4:**
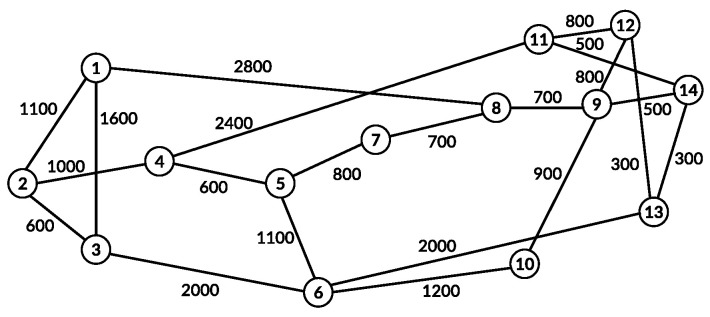
The NSFNET topology with 14 nodes and 21 links.

**Figure 5:**
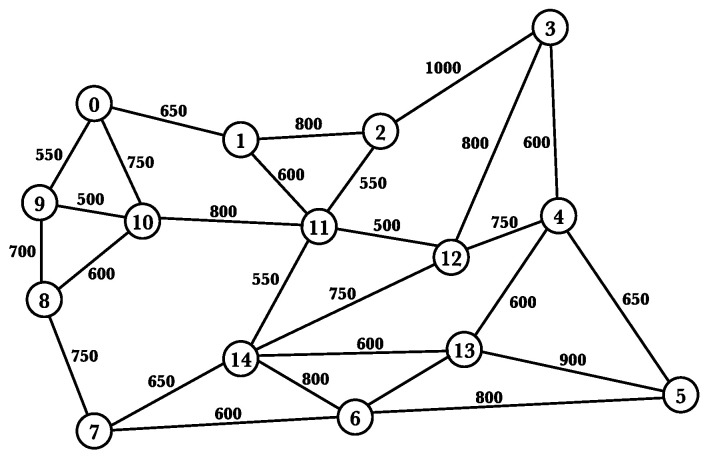
The CHNNET topology with 15 nodes and 27 fiber links.

We considered the NSFNET and CHNNET topologies. The NSFNET topology (Figure 4) has 14 nodes and 21 optical fiber links, and the CHNNET topology (Figure 5) has 15 nodes and 27 optical fiber links.

We employed the FlexGrid simulator [45] to simulate the scenarios used in the evaluation of CO-OP framework. The virtualization module in the simulator (available at https://bit.ly/2OKwwWI, accessed on 2 November 2021)) is based on the set of information described in Reference [10], and VON requests arrive according to a Poisson process. We assume that the computing resources of each physical node are 100, and each physical link has seven cores, with 320 frequency slots [44]. For each VON request, the number of virtual nodes is randomly determined by a uniform distribution in the interval [3,N−1], and pairs of virtual nodes are randomly connected with a probability 0.5 [10]. The number of alternative physical nodes $A_{l}^{m}$ for $n_{v}^{m_{i}}$ is chosen using a uniform distribution in the interval [2, 4], and the computing resources requirement $c_{v}^{m_{i}}$ is also chosen using a uniform distribution in the interval [1, 4] [10]. The request capacity of each connection is chosen using a uniform distribution between 50 Gb to 400 Gb [44]. The number of candidates paths, *K*, for the KSP algorithm was set to 3.

We used the Bandwidth Blocking Ratio (BBR), acceptance ratio, and mean crosstalk. For the evaluation of the supervised algorithm in the monitor and analyze functions, we performed tests using all possible combinations of algorithms in Table 3. In this paper, we display the most representative results that illustrate the benefits of using CO-OP. Different versions of CO-OP employed different combinations of algorithms. In CO-OP I, both the monitor and analyze functions employ the Random Forest algorithm because it produces the best overall result. In CO-OP II, the monitor function employs the Random Forest algorithm, while the analyze function employs Neural Networks. In CO-OP III, both the monitor and analyze functions employ a Neural Network, while, in CO-OP IV, they use the Support Vector Machine (SVM) algorithm. The performance of the CO-OP variations is plotted as a function of the number of VON requests in the simulation.

**Figure 6:**
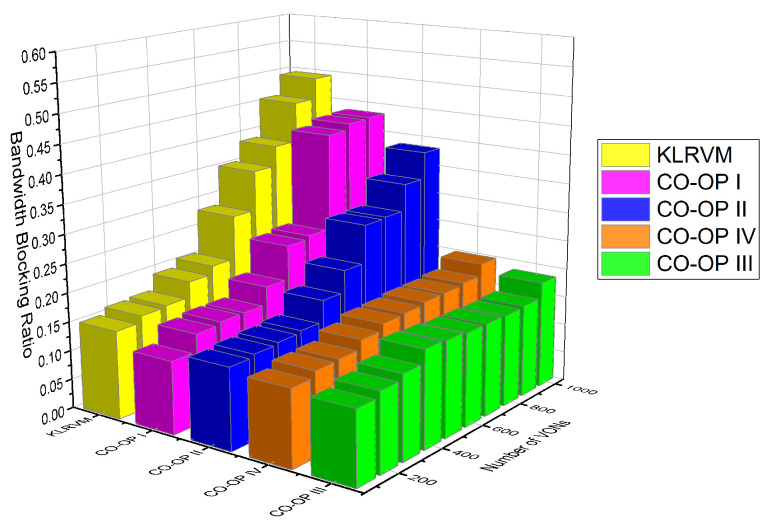
BBR for the NSFNET topology.

Figure 6 shows the BBR for the NSFNET topology. The KLRVM algorithm produced BBR values 20% higher than those produced by the CO-OP IV for $3\times 10^{3}$ VONs. The KLRVM algorithm produced BBR values 50% greater than those produced by the CO-OP III for $6\times 10^{3}$ VONs. The CO-OP I produced BBR values two orders of magnitude greater than those produced by the CO-OP III for $7\times 10^{3}$ VONs. The KLRVM algorithm produced BBR values 40% greater than those produced by both CO-OP III and CO-OP IV for $10^{4}$ VONs. These BBR results obtained by employing the CO-OP framework demonstrate its outstanding performance under heavy traffic load.

**Figure 7:**
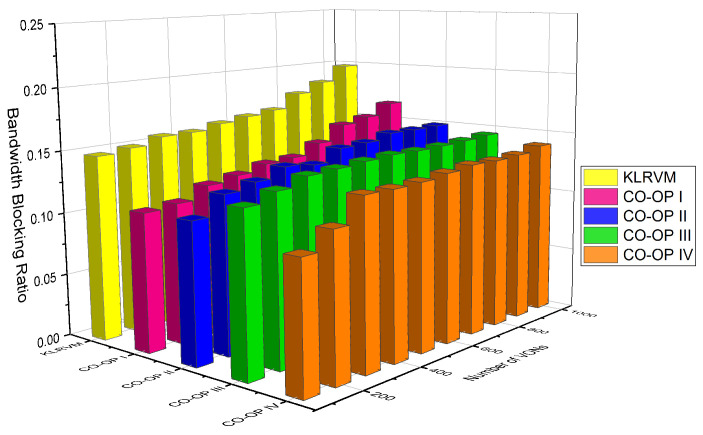
BBR results for the CHNNET topology.

Figure 7 shows the BBR results for the CHNNET topology. The KLRVM algorithm produced BBR values 15% greater than those produced by CO-OP II for $3\times 10^{3}$ VONs. The CO-OP III produced BBR values 18% lower than those produced by the KLRVM algorithm for $7\times 10^{3}$ VONs. The CO-OP IV produced BBR values 28% lower than those produced by the KLRVM algorithm for $10^{4}$ VONs. The BBR values results differ depending on the topology due to the connectivity degree. Lower BBR improvements were achieved since the CHNNET topology has a higher node connectivity degree than the NSFNET topology. CO-OP III and CO-OP IV consistently produced lower BBR than KLRVM and stayed lower than 30% across all simulations and lower than 50% across all CO-OP versions. Moreover, values of BBR greater than 20% for heavily loaded systems in order to evaluate the proposal under extreme conditions. Under non-extreme conditions, BBR values were below 20%, which are acceptable values.

**Figure 8:**
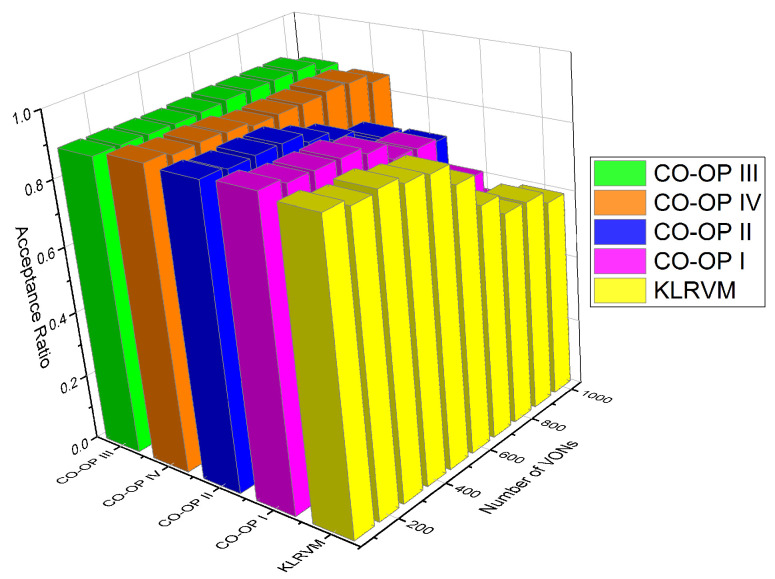
Acceptance ratio for the NSFNET topology.

Figure 8 shows the acceptance ratio for the NSFNET topology. The KLRVM algorithm produced acceptance ratio values 5% lower than those produced by the CO-OP III for $4\times 10^{3}$ VONs. The CO-OP IV produced acceptance ratio values 28% greater than those produced by the KLRVM algorithm for $7\times 10^{3}$ VONs. The KLRVM algorithm produced acceptance values 15 lower than those produced by the CO-OP I for $7\times 10^{3}$ VONs. The CO-OP III produced acceptance ratio values 30% larger than those produced by the KLRVM algorithm considering $10^{4}$ VONs. In general, the CO-OP III produced the highest acceptance values compared to the other algorithms. The acceptance ratio values show that a higher number of VONs can be established when CO-OP is employed. CO-OP allows the acceptance of VON with large bandwidth demands than do KLRVM. Moreover, the fragmentation ratio also decreased when the CO-OP framework was used.

**Figure 9:**
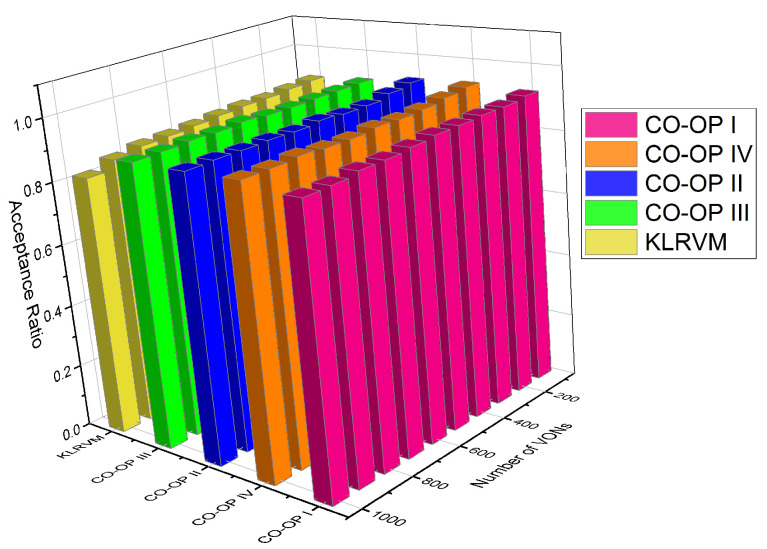
Acceptance ratio for the CHNNET topology.

Figure 9 shows the acceptance ratio for the CHNNET topology. In most of the scenarios, the acceptance ratio values obtained by CO-OP were higher than those produced by the KLRVM algorithm. However, the values for this topology were very similar. The KLRVM algorithm produced acceptance ratio values 4% lower than those produced by the CO-OP III with $2\times 10^{3}$ VONs. The acceptance ratio values produced by the CO-OP III and CO-OP IV were 5% greater than those produced by the KLRVM algorithm with $10^{4}$ VONs. The acceptance ratio values did not differ much as a function of the learning algorithm considered. Comparing with the results for the NSF topology, it is possible to see that, when the topology has a high node degree, more requests are accepted, resulting in greater acceptance of VONs, regardless of the type of learning algorithm that the CO-OP used.

**Figure 10:**
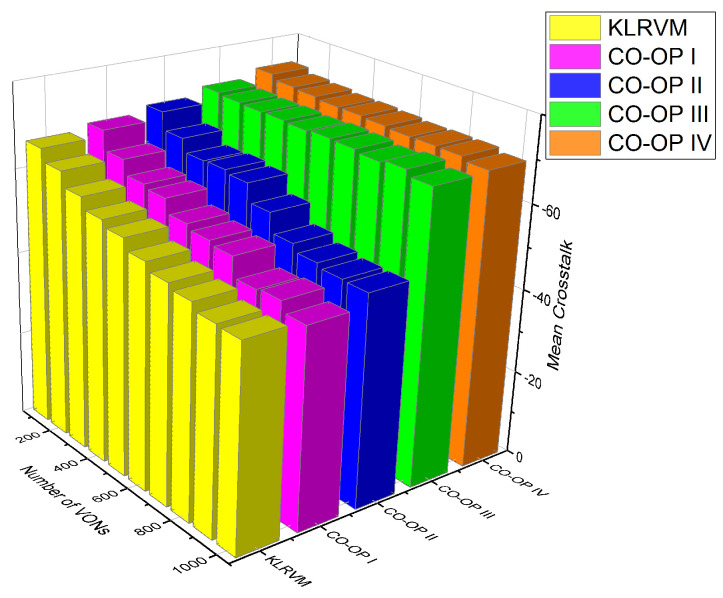
Mean crosstalk for the NSFNET topology.

Figure 10 shows the mean crosstalk values for the NSFNET topology. The crosstalk values produced by CO-OP IV and CO-OP III were similar and higher than those produced by the other CO-OP variations for most loads. CO-OP III produced crosstalk values 16% lower than those produced by the KLRVM algorithm for $4\times 10^{3}$ VONs. CO-OP IV produced crosstalk values 13% lower than those produced by the CO-OP II for $5\times 10^{3}$ VONs. The KLRVM algorithm produced crosstalk values 31% higher than those produced by the CO-OP IV for $10^{4}$ VONs. The crosstalk values demonstrate the efficiency of employing the CO-OP framework. In most simulations, the crosstalk values were lower than the ones produced by the KLRVM algorithm. Lower crosstalk values indicate that the quality of transmission is higher when CO-OP is employed. The mean crosstalk values have the same behavior as the BBR, showing a correlation between blocking and crosstalk interference. Crosstalk values are acceptable according to the interference tolerance reported in Reference [32], used in this paper.

**Figure 11:**
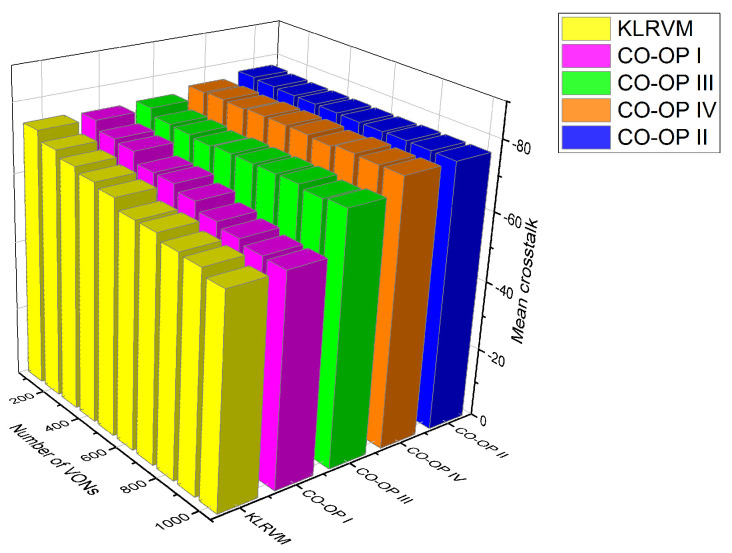
Mean crosstalk for the CHNNET topology.

Figure 11 shows the mean crosstalk results for the CHNNET topology. The crosstalk values produced by CO-OP IV was the lowest for most of the loads. The KLRVM algorithm produced crosstalk values 8% higher than those produced by the CO-OP IV for $3\times 10^{3}$ VONs. The CO-OP I produced crosstalk values 15% higher than those produced by CO-OP II for $6\times 10^{3}$ VONs. The KLRVM algorithm produced crosstalk values 21% higher than those produced by CO-OP IV for $10^{4}$ VONs. The crosstalk values were significantly lower when compared to those produced when employing the KLRVM algorithm for the two topologies, meaning that the CO-OP framework creates effective plans which result in allocations with better QoT.

## 7. Conclusions

In this paper, we introduced the CO-OP framework, to improve the configuration of VONs. The framework is based on MAPE functions using learning techniques to classify statistics from the network to identify potential problems. If a problem is identified, the CO-OP creates a plan using a *Q*-Learning algorithm, and then a centralized control plane is responsible for executing the plan.

The results show that the use of CO-OP can decrease BBR up to 30% and VON acceptance by 50%, and both impact the QoS provided. The use of CO-OP can also reduce 50% of the crosstalk produced, resulting in better QoT compared to that given by traditional VON configuration algorithms.

In the future, we intend to develop a testbed to evaluate the proposed framework. To set a testbed, we need to obtain real-world datasets of EON-SDM networks. We then need to identify a set of features before the experimental operation of the network. The selected features can differ from the ones presented in this paper since the most significant features depend on the specific dataset used. To select features, we can employ feature selection algorithms and also use specialist knowledge. After that, specific supervised learning algorithms need to be selected for the monitor and analyze functions in the training process. To select them, we can consider different metrics, such as classification accuracy, logarithmic loss, F1 score, and mean square error, depending on the learning algorithms employed. In this paper, we used only the classification accuracy to evaluate the training process.

After training the supervised models for the monitor and analyze functions, we should determine a continuous process to update the models to improve network performance. The updates can follow two criteria: the network metrics or data volume. We need to continuously capture data from the network operation and re-train the model to make the update. As an optimization, we can compress messages sent, as well as received, to decrease communication costs.

CO-OP will need to be implemented and integrated with a central control plane in real operational networks. In this paper, we consider the control plane, management plane, and knowledge plane. Other metrics can also be considered, such as energy efficiency, energy consumption, fragmentation ratio, and cost, allowing a thorough comparison with traditional management schemes to evaluate CO-OP performance.

In the testbed, dynamically adapting the number of interactions with the control plane can be a challenging task. Moreover, communication security can also be challenging since the data collected from the virtual and physical networks can interfere with the network performance. Moreover, the testbed can employ different Quantum Key Distribution (QKD) schemes to increase security in inter-plane communications. 

## Figures and Tables

**Table 1:** Existing solutions in the literature.

References	Year	Main Achievement
[10]	2012	Heuristic algorithm to configure VONs over single-mode-fiber optical networks.
[21]	2017	Algorithm to enhance load balance among network links and reduce spectrum usage.
[16,17]	2016–2017	Load-balancing algorithms were introduced for virtual network configuration on physical networks employing MCFs and FMFs.
[19]	2017	Algorithm for virtual network topology adaptation using artificial neural networks including neural networks.
[22,23]	2018 and 2021	Virtual network reconfiguration algorithms for optical networks with single-mode fibers.
[18]	2020	Algorithms to cope with the spectrum fragmentation in lightpath establishment for VONs.
[24,25]	2020	Machine learning algorithms to handle the spectrum fragmentation problem.
[26]	2020	Distributed SDN control system with blockchain technology.

**Table 2:** Notation used in the paper.

Symbol	Description
*V*	Set of physical nodes, where $V=\{v_{1},v_{2},\ldots ,v_{N}\}$.
*E*	Set of physical links, where $E=\{e_{1},e_{2},\ldots ,e_{N_{l}}\}$.
*G*	G=(V,E) represents the physical network.
$N_{l}$	Number of physical links.
*N*	Number of physical nodes.
*F*	$F=\{f_{1,1},f_{1,2},\ldots ,f_{|F|,|F|}\}$ represents the possibilities of usage of the network physical link spectrum to allocate the requested bandwidth.
$G_{v}$	$G_{v}=\left(V_{v},E_{v}\right)$ represents the current virtual network.
$V_{v}$	$V_{v}=\left\{v_{v}^{1},v_{v}^{2},\ldots ,v_{v}^{|V_{v}|}\right\}$ represents a set of virtual requests that have allocated resources on the physical network
$E_{v}$	$E_{v}=\left\{e_{v}^{1},e_{v}^{2},\ldots ,v_{e}^{|E_{v}|}\right\}$ represents a set of virtual links.
*C*	Set of compute resource required, where $C=\{c_{1},c_{2},\ldots \}$ represents a set of computing resources in $e_{i,j}$, where ∣C∣ is the number of computing resources required by $G_{v}$.

**Table 3:** Accuracy of supervised algorithms when used by monitor and analyze functions.

Classifier	Description	Accuracy of Monitor (%)	Accuracy of Analyze (%)
Random Forest	Random Forest classifier combines random variable choice at nodes and bootstrap aggregation. For training decision trees, it uses a subset of the dataset.	95	90
*K*-Nearest Neighbors	This classifier similar samples into *k* groups.	90	95
Naive Bayes	This classifier is a probabilistic machine learning model based on Bayes theorem.	95	97.5
Logistic Regression	This classifier is a statistical model that in its basic form uses a logistic function to model a binary dependent variable, although many more complex extensions exist.	90	90
Neural Networks	Neural networks represent an attempt to mimic the biological nervous system for both architecture and information processing strategies.	90	90
Support Vector Machine	This linear model for classification or regression can create a line or a hyper plane which separates the data into classes.	90	97.8

**Table 4:** Possible actions given by the *Q*-Learning algorithm.

Action	Application	Associated Problem	Type
Reconfiguration	Link	Spectrum inefficiency	Medium
Redirection	Link	Unbalanced load	High
Blocking	Link	Unbalanced or spectrum inefficiency	High
Blocking	Node	Cost	High
Limiting	Link	Unbalanced, spectrum inefficiency, or overload	Medium
Limiting	Node	Cost	Medium
Nothing	Node or link	No problem was identified	Low

## Data Availability

Not applicable.

## References

[1] Jinno M., Takara H., Kozicki B., Tsukishima Y., Sone Y., Matsuoka S. (2009). Spectrum-efficient and scalable elastic optical path network: Architecture, benefits, and enabling technologies. Commun. Mag. IEEE.

[2] Gong L., Zhou X., Liu X., Zhao W., Lu W., Zhu Z. (2013). Efficient resource allocation for all-optical multicasting over spectrum-sliced elastic optical networks. J. Opt. Commun. Netw..

[3] Zhu Z., Lu W., Zhang L., Ansari N. (2012). Dynamic service provisioning in elastic optical networks with hybrid single-/multi-path routing. J. Light. Technol..

[4] Yin Y., Zhang H., Zhang M., Xia M., Zhu Z., Dahlfort S., Yoo S.J.B. (2013). Spectral and Spatial 2D Fragmentation-Aware Routing and Spectrum Assignment Algorithms in Elastic Optical Networks. J. Opt. Commun. Netw..

[5] Richardson D., Fini J., Nelson L.E. (2013). Space-division multiplexing in optical fibres. Nat. Photonics.

[6] Saridis G.M., Alexandropoulos D., Zervas G., Simeonidou D. (2015). Survey and evaluation of space division multiplexing: From technologies to optical networks. IEEE Commun. Surv. Tutorials.

[7] Thyagaturu A.S., Mercian A., McGarry M.P., Reisslein M., Kellerer W. (2016). Software defined optical networks (SDONs): A comprehensive survey. IEEE Commun. Surv. Tutorials.

[8] Li S., Han K., Huang H., Sun Q., Liu J., Zhao S., Zhu Z. (2017). SR-PVX: A source routing based network virtualization hypervisor to enable POF-FIS programmability in vSDNs. IEEE Access.

[9] Rodriguez E., Alkmim G., Batista D.M., da Fonseca N.L. Trade-off between bandwidth and energy consumption minimization in virtual network mapping. Proceedings of the 2012 IEEE Latin-America Conference on Communications.

[10] Wang Z., Han Y., Lin T., Tang H., Ci S. Virtual network embedding by exploiting topological information. Proceedings of the 2012 IEEE Global Communications Conference (GLOBECOM).

[11] Gong L., Jiang H., Wang Y., Zhu Z. (2016). Novel location-constrained virtual network embedding LC-VNE algorithms towards integrated node and link mapping. IEEE/ACM Trans. Netw..

[12] Rodriguez E., Alkmim G.P., da Fonseca N.L., Batista D.M. (2015). Energy-aware mapping and live migration of virtual networks. IEEE Syst. J..

[13] Gong L., Zhu Z. (2013). Virtual optical network embedding (VONE) over elastic optical networks. J. Light. Technol..

[14] Alkmim G.P., Batista D.M., da Fonseca N.L.S. Optimal mapping of virtual networks. Proceedings of the 2011 IEEE Global Telecommunications Conference-GLOBECOM 2011.

[15] Brasileiro Í, Costa L., Drummond A. (2020). A survey on challenges of Spatial Division Multiplexing enabled elastic optical networks. Opt. Switch. Netw..

[16] Huang H., Huang S., Yin S., Zhang M., Zhang J., Gu W. (2016). Virtual Network Provisioning Over Space Division Multiplexed Optical Networks Using Few-Mode Fibers. J. Opt. Commun. Netw..

[17] Xuan H., Wang Y., Xu Z., Hao S., Wang X. (2017). Virtual optical network mapping and core allocation in elastic optical networks using multi-core fibers. Opt. Commun..

[18] Zhu R., Li S., Wang P., Yuan J. (2020). Time and spectrum fragmentation-aware virtual optical network embedding in elastic optical networks. Opt. Fiber Technol..

[19] Morales F., Ruiz M., Gifre L., Contreras L.M., López V., Velasco L. (2017). Virtual Network Topology Adaptability Based on Data Analytics for Traffic Prediction. J. Opt. Commun. Netw..

[20] Mestres A., Rodriguez-Natal A., Carner J., Barlet-Ros P., Alarcón E., Solé M., Muntés-Mulero V., Meyer D., Barkai S., Hibbett M.J. (2017). Knowledge-Defined Networking. SIGCOMM Comput. Commun. Rev..

[21] Zhao G., Xu Z., Ye Z., Wang K., Wu J. A load balancing algorithm based on key-link and resources contribution degree for virtual optical networks mapping. Proceedings of the 2017 International Conference on Computer, Information and Telecommunication Systems (CITS).

[22] Rafique D., Velasco L. (2018). Machine Learning for Network Automation: Overview, Architecture, and Applications[Invited Tutorial]. J. Opt. Commun. Netw..

[23] Pan X., Zhao S., Yang H., Tang S., Zhu Z. (2021). Scheduling Virtual Network Reconfigurations in Parallel in Hybrid Optical/Electrical Datacenter Networks. J. Light. Technol..

[24] Wei W., Gu H., Pattavina A., Wang J., Zeng Y. (2020). Optimizing energy and spectrum efficiency of virtual optical network embedding in elastic optical networks. Opt. Switch. Netw..

[25] Trindade S., da Fonseca N.L. (2021). Machine Learning for Spectrum Defragmentation in Space-Division Multiplexing Elastic Optical Networks. IEEE Netw..

[26] Ding S., Shen G., Pan K.X., Bose S.K., Zhang Q., Mukherjee B. (2020). Blockchain-Assisted Spectrum Trading Between Elastic Virtual Optical Networks. IEEE Netw..

[27] Ohba T., Arakawa S., Murata M. (2016). Virtual network reconfiguration in elastic optical path networks for future bandwidth allocation. J. Opt. Commun. Netw..

[28] da Silva I.R., Xavier E.C., da Fonseca N.L. Algorithms for selection and allocation of virtual network requests. Proceedings of the 2013 IEEE Latin-America Conference on Communications.

[29] Alkmim G.P., Batista D.M., Da Fonseca N.L. (2013). Mapping virtual networks onto substrate networks. J. Internet Serv. Appl..

[30] Chatterjee B.C., Fadini W., Oki E. (2016). A spectrum allocation scheme based on first–last-exact fit policy for elastic optical networks. J. Netw. Comput. Appl..

[31] Freeman L.C. (1978). Centrality in social networks conceptual clarification. Soc. Netw..

[32] Hayashi T., Taru T., Shimakawa O., Sasaki T., Sasaoka E. (2011). Design and fabrication of ultra-low crosstalk and low-loss multi-core fiber. Opt. Express.

[33] Dobson S., Denazis S., Fernández A., Gaïti D., Gelenbe E., Massacci F., Nixon P., Saffre F., Schmidt N., Zambonelli F. (2006). A survey of autonomic communications. ACM Trans. Auton. Adapt. Syst. (TAAS).

[34] Kliazovich D., Granelli F., Da Fonseca N.L. (2010). Architectures and Cross-Layer Design for Cognitive Networks.

[35] Kliazovich D., Granelli F., Fonseca N., Bouvry P. (2013). Architectures and Information Signaling Techniques for Cognitive Networks. Evolution of Cognitive Networks and Self-Adaptive Communication Systems.

[36] Sakly H. (2020). Self-Organization and Autonomous Network Survey. Int. J. Netw. Secur. Its Appl. (IJNSA).

[37] Ayoubi S., Limam N., Salahuddin M.A., Shahriar N., Boutaba R., Estrada-Solano F., Caicedo O.M. (2018). Machine learning for cognitive network management. IEEE Commun. Mag..

[38] Watkins C.J., Dayan P. (1992). Q-learning. Mach. Learn..

[39] Dearden R., Friedman N., Russell S. (1998). Bayesian Q-Learning.

[40] van Hasselt H., Wiering M., van Otterlo M. (2012). Reinforcement Learning in Continuous State and Action Spaces. Reinforcement Learning: State-of-the-Art.

[41] Rummery G.A., Niranjan M. (1994). On-Line Q-Learning Using Connectionist Systems.

[42] Pedregosa F., Varoquaux G., Gramfort A., Michel V., Thirion B., Grisel O., Blondel M., Prettenhofer P., Weiss R., Dubourg V. (2011). Scikit-learn: Machine Learning in Python. J. Mach. Learn. Res..

[43] MacGlashan J., Michael Littman S.T., desJardins M. Brown-UMBC Reinforcement Learning and Planning (BURLAP). https://github.com/jmacglashan/burlap.

[44] Trindade S., da Fonseca N.L.S. Proactive Fragmentation-Aware Routing, Modulation Format, Core, and Spectrum Allocation in EON-SDM. Proceedings of the ICC 2019—2019 IEEE International Conference on Communications (ICC).

[45] Moura P.M., André D.M., Trindade S. (2018). FlexGridSim: Flexible Grid Optical Network Simulator. https://www.lrc.ic.unicamp.br/FlexGridSim/.

